# Supplementary material for: Inflammatory factors and risk of lung adenocarcinoma: a Mendelian randomization study mediated by blood metabolites
Source: Front Endocrinol (Lausanne). 2024 Aug 27;15:1446863. doi: 10.3389/fendo.2024.1446863 (PMC11384989; doi:10.3389/fendo.2024.1446863)
Supplement: Supplementary file 1 [file DataSheet1.zip › supplementary files/S2/GCST90200922/funnelplot.pdf]

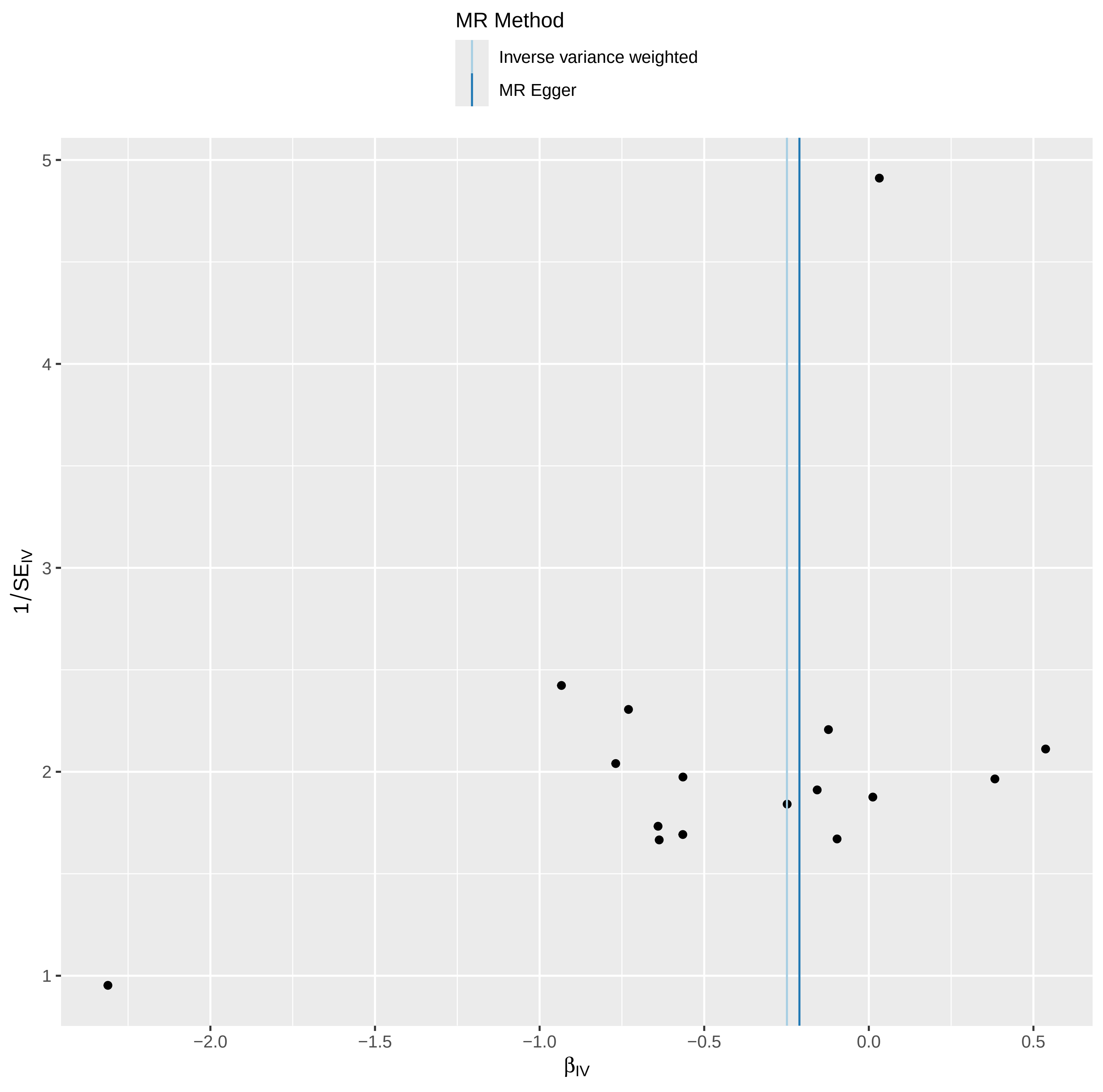

MR Method
Inverse variance weighted
MR Egger
5
4
3
2
1
1/SE_IV
−2.0
−1.5
−1.0
−0.5
0.0
0.5
β_IV
